# A Small Fraction of Progenitors Differentiate Into Mature Adipocytes by Escaping the Constraints on the Cell Structure

**DOI:** 10.3389/fcell.2021.753042

**Published:** 2021-10-11

**Authors:** Mahmoud Ahmed, Trang Huyen Lai, Deok Ryong Kim

**Affiliations:** Department of Biochemistry and Convergence Medical Sciences, Institute of Health Sciences, Gyeongsang National University School of Medicine, Jinju, South Korea

**Keywords:** deconvolution, differentiation, adipocytes, 3T3-L1, subpopulation analysis

## Abstract

Differentiating 3T3-L1 pre-adipocytes are a mixture of non-identical culture cells. It is vital to identify the cell types that respond to the induction stimulus to understand the pre-adipocyte potential and the mature adipocyte behavior. To test this hypothesis, we deconvoluted the gene expression profiles of the cell culture of MDI-induced 3T3-L1 cells. Then we estimated the fractions of the sub-populations and their changes in time. We characterized the sub-populations based on their specific expression profiles. Initial cell cultures comprised three distinct phenotypes. A small fraction of the starting cells responded to the induction and developed into mature adipocytes. Unresponsive cells were probably under structural constraints or were committed to differentiating into alternative phenotypes. Using the same population gene markers, similar proportions were found in induced human primary adipocyte cell cultures. The three sub-populations had diverse responses to treatment with various drugs and compounds. Only the response of the maturating sub-population resembled that estimated from the profiles of the mixture. We then showed that even at a low division rate, a small fraction of cells could increase its share in a dynamic two-populations model. Finally, we used a cell cycle expression index to validate that model. To sum, pre-adipocytes are a mixture of different cells of which a limited fraction become mature adipocytes.

## 1. Introduction

Cell cultures contain populations of non-identical cells. Early on, pre-adipocytes cell cultures were recognized to be heterogeneous. Green *et al*. described the heterogeneous nature of differentiating 3T3-L1 cell (Green and Kehinde, 1975). Lee et al. (2019) showed that adipocyte progenitors within a single fat depot give rise to functionally distinct adipocytes. Cell cultures never attain a 100% differentiation efficiency; thus, only a fraction of the pre-adipocytes are responsive. A key question is how large or small this fraction is. Loo *et al*. showed that a minority of pre-adipocytes gives rise to mature adipocytes with characteristic gene expression and lipid droplets (Loo et al., 2009).

The varying cellular response to the induction media could be because of their intrinsic properties or is a stochastic process. Stochasticity and cell-cell interactions were suggested as key contributors to the differentiation of stem cells (Smith et al., 2015). If the pre-adipocytes are intrinsically different, we should be able, in principle, to find mechanisms that confer responsiveness/resistance to the sub-populations. A study proposed that the adipocyte's ability to mature and accumulate fat is heritable. Modifying the key adipogenic transcription factor peroxisome proliferator-activated receptor gamma (*Pparg*) promoter region marks the cell capacity to differentiate, and re-differentiate (Katz et al., 2014).

Differentiation is a transcriptional and morphological development event. Cells proliferate only for 1 day after the 3T3-L1 induction in what is known as post-confluent mitosis followed by growth arrest (Bernlohr et al., 1985). Cell division and DNA replication are necessary to allow transcription factors into the open chromatin. This, in turn, drives the development of the new phenotype. Within days, the cell accumulates lipids in droplets and remodels its interior into a distinct fat cell.

Changes in the cytoskeleton and extracellular matrix rarely feature during the study of adipocyte differentiation. This is unfortunate given their prime importance to the morphological development of the adipocytes. It is clear from research done in stem cells with differentiation potentials; they have to respond to extracellular signals, not only by modulating the gene expression but their internal structure (Yourek et al., 2007; Guilak et al., 2009). Yang et al. (2013) showed that remodeling the cytoskeleton through the acetylation of alpha-tubulin is necessary for the differentiation of pre-adipocytes. Moreover, it was even suggested that actin cytoskeleton might interfere with the transcriptional regulation of the differentiation process by controlling the translocation of antagonistic nuclear factors (Nobusue et al., 2014).

Profiling by RNA-Seq is an efficient way to study gene expression changes between two or more conditions. However, the expression signal, in this case, is averaged over a mixed population of various cell types. Single-cell RNA-Seq (scRNA-Seq) is the most straightforward way to test the gene expression at the level of the individual cells. Because these datasets are not always available, deconvolution of bulk RNA-Seq data can be an alternative. In this study, the sub-populations in the mixture were estimated from their gene signatures and studied separately.

Convex analysis of mixtures (CAM) is an unsupervised method to deconvolute the profiles of samples from mixed phenotypes into their constituents (Wang et al., 2016). The creators of the method applied it to a dataset of gene expression in yeast at different cell cycle stages. CAM could detect a composite of sub-populations consistent with the true labels. In addition, the sub-populations gene markers identified by CAM were related to the corresponding phases. Herrington et al. (2018) used this method to derive distinct phenotypes in arterial tissues. Classification of the cell types based on their markers was consistent with tumor necrotic factor (TNF) alpha pathway activation.

Here we describe deconvoluting the gene expression of differentiating 3T3-L1 pre-adipocytes from bulk RNA-Seq data. The goal is to determine the distinct phenotypes in the cell cultures and estimate their fractions. Then we showed which of these fractions responded to the induction stimuli and became mature adipocytes. Finally, we attempted to explain the differences between the sub-populations as to which functions their specific markers involve in, which type of cells they give rise to at the end of the differentiation course, and their response to treatment with various drugs and compounds.

## 2. Materials and Methods

### 2.1. Cell Culture and Differentiation

3T3-L1 is a mouse pre-adipocyte cell line that can be induced to differentiate into mature adipocytes when treated with a chemical cocktail. A chemical cocktail of 1-Methyl-3-isobutylxanthine, Dexamethasone and Insulin (MDI) is known to induce the differentiation (Green and Kehinde, 1975). The treatment starts with a fully confluent 3T3-L1 cell culture (pre-adipocyte), accumulating lipid into lipid droplets within days. The time course comprises three stages; day 2–4, early; up to day 7 intermediate; and up to 14 days is the late-differentiation stage. A similar protocol produces a comparable differentiation course in human primary pre-adipocytes.

### 2.2. Data Curation and Processing

#### 2.2.1. RNA-Seq Data

We surveyed GEO and SRA repositories for high-throughput sequencing data of MDI-induced 3T3-L1 and human primary pre-adipocyte samples at different time points (Table 1). Ninety-eight RNA-Seq samples were included in the study. This dataset was previously curated, and the processed data was packaged in a Bioconductor experimental data package curatedAdipoRNA (Ahmed and Kim, 2019). Briefly, raw reads were downloaded from the SRA ftp server using Wget. FASTQC was used to assess the quality of the raw reads (Andrews, 2020). For RNA-Seq, raw reads were aligned to UCSC mm10 mouse genome using HISAT2 (Kim et al., 2015). FeatureCounts was used to count the aligned reads (bam) in known genes (Liao et al., 2014).

**Table 1:** RNA-Seq datasets of differentiating 3T3-L1 cells.

**SRA ID**	**Time (h)**	**Ref**.
SRP010905	192	Lo et al., 2013
SRP029602	–48–144	Al Adhami et al., 2015
SRP029985	0–168	Duteil et al., 2014
SRP033778	–48–240	Zhao et al., 2014
SRP041765	0–4	Siersbæk et al., 2014
SRP045772	0–48	Lim et al., 2015
SRP051775	168	Buchner et al., 2015
SRP066991	–96–168	Brunmeir et al., 2016
SRP078506	0–48	Brier et al., 2017
SRP090166	0–168	Park et al., 2017
SRP092793	240	Chaudhary et al., 2016
SRP100171	0–192	Chen et al., 2018
SRP100871	0–168	Siersbæk et al., 2017
SRP102079	0–4	Ryu et al., 2018
SRP109255	–48–24	Maroni et al., 2017
SRP119259	192	You et al., 2017

#### 2.2.2. Microarray Data

We conducted a similar survey of the literature for studies that subject the same cell line model for chemical or genetic perturbations (Table 2). Forty-two microarray of mature adipocytes (> 7 days post MDI-induction) treated with thiazolidinediones (TZD), four short-chain fatty acids (SFA), or three nonsteroidal anti-inflammatory drugs (NSAID) were previously collected and curated. The processed data were packaged in another experimental data package curatedAdipoArray (Ahmed et al., 2020). In addition, 24 microarray samples of MDI-induced human primary pre-adipocytes were downloaded from GEO in the form of processed probe intensities (GSE98680) (Verbanck et al., 2017).

**Table 2:** Microarray datasets of differentiating 3T3-L1 under pharmacological perturbations.

**GEO ID**	**Type**	**Treatment**	**Ref**.
GSE1458	TZD	pioglitazone/ rosiglitazone/ troglitazone	Hsiao et al., 2004
GSE42220	SFA	oleic acid/palmitic acid/palmitoleic acid/stearic acid	Shaw et al., 2013
GSE64075	NSAID	diclofenac/ ibuprofen/ indomethacin	Puhl et al., 2015

Microarray data were downloaded from GEO in the form of probe intensities. Probe metadata (gene symbols) were used to collapse the intensities into gene level using the maximum average (Horvath and Dong, 2008). Data were normalized using normalize between arrays, and batch effects were removed using empirical Bayesian adjustments (Ritchie et al., 2015; Leek et al., 2020).

### 2.3. Identifying Adipocyte Populations

**Figure 1:**
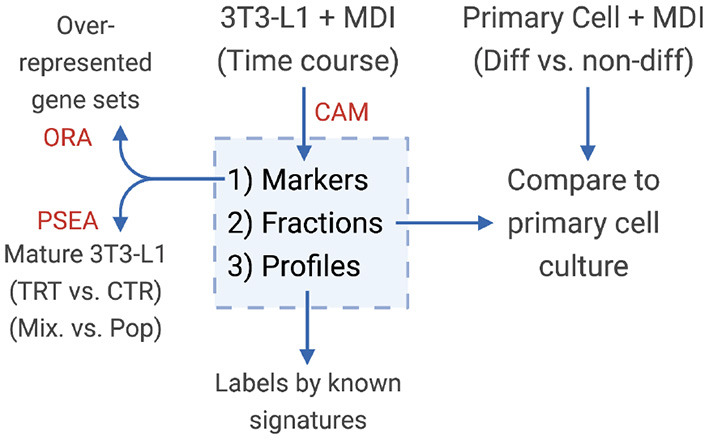
Diagram of the study design. Convex analysis of mixtures (CAM) was applied on MDI-induced 3T3-L1 time-course gene expression to estimate the sub-population (1) markers (2) fractions, and (3) specific expression profiles. Markers were tested for over-representation in gene sets (ORA). Sub-population fractions were validated in primary adipocytes, and expression profiles were examined for known gene signatures.

Convex Analysis of Mixtures (CAM) was used to deconvolute gene counts expression profiles (Wang et al., 2016). This analysis was applied using debCAM (Chen, 2020). Briefly, the scatter simplex of the cell mixture is a rotated and compressed version of the scatter simplex of the pure sub-populations (Figure 1). The sub-populations and their markers reside at the extremities of the shape. The one-vs.-everyone fold-change was calculated for all genes and used to select gene markers. Using the expression of the markers, the relative fractions of the sub-populations in the mixture were estimated by standardized averaging. The estimated fractions were used to deconvolute the mixed expressions into sub-populations specific profiles by least-square regressions.

### 2.4. Validating Markers and Populations

We validated the analysis using permutations of the identified markers and an independent dataset of gene expression profiles from primary adipocyte. To isolate the effect of the gene markers' set size and outliers, the fractions of the sub-populations were re-estimated using a smaller set chosen at random from the identified markers. The set size was chosen to be equal to that of the smallest sub-population. The same process was repeated several times for each perumutation of the markers. A supervised version of CAM was applied using the expression profiles of human primary adipocytes using the full set of markers.

### 2.5. Gene Set Over-Representation

Lists of sub-populations specific markers were annotated using the gene ontology terms (org.Mm.eg.db) and tested for over-representation using ClusterProfiler (Yu et al., 2012; Carlson, 2020). For each gene set, the observed fraction of gene in the list was compared to the fraction expected by chance. *P*-values were calculated for each gene set and adjusted for multiple testing using false discovery rate (FDR).

### 2.6. Differential Expression Analysis

The effect of drugs and compounds treatment on gene expression of mature adipocytes was evaluated at the mixture and population levels. The differential gene expression between treatment and control groups was calculated using LIMMA (Ritchie et al., 2015). Population-specific gene expression analysis was applied to the same comparisons using PSEA (Kuhn et al., 2011). The previously identified markers of the three sub-populations were used to construct a reference population and perform one-to-one comparisons. *P*-values were calculated for each gene and adjusted for multiple testing using FDR.

### 2.7. Software and Reproducibility

The analysis was conducted in an R and using Bioconductor packages (Huber et al., 2015; R Core Team, 2020). The software environment is available as a docker image (https://hub.docker.com/r/bcmslab/adipo_deconv). The code for reproducing the figures and tables in this document is open-source (https://github.com/BCMSLab/adipo_deconv).

## 3. Results

### 3.1. Pre-adipocytes Cell Cultures Consist of Three Distinct Sub-populations

**Figure 2:**
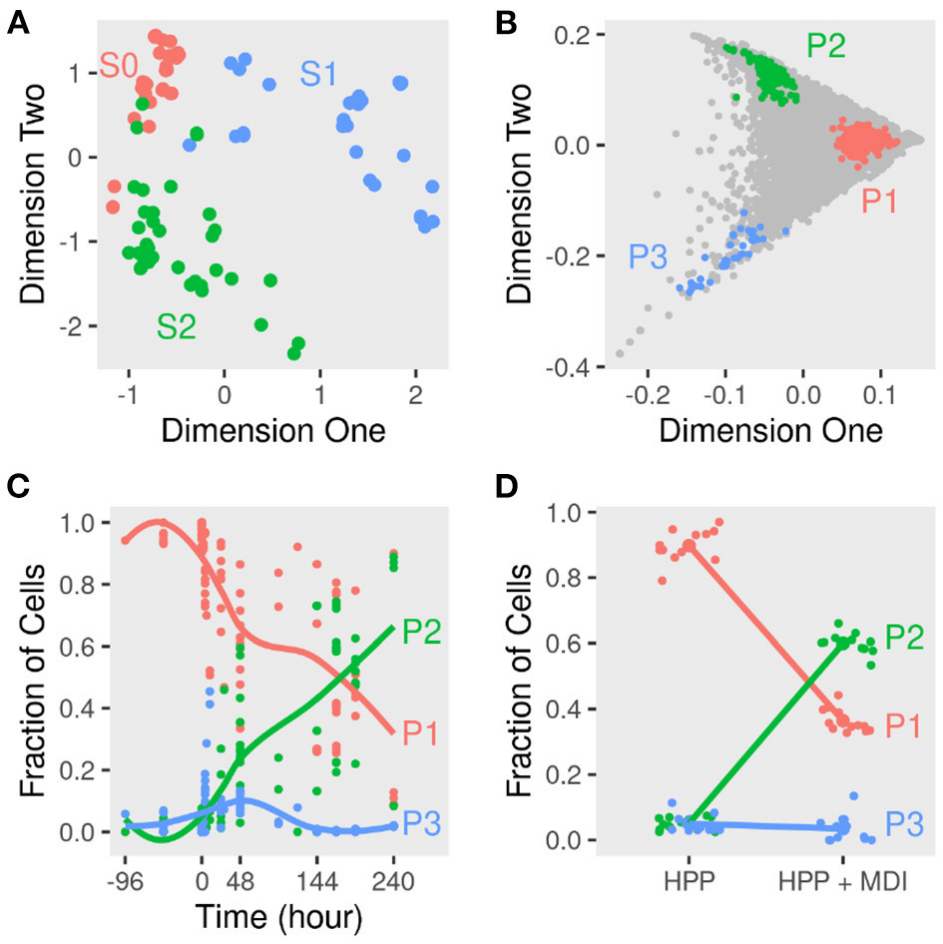
Dimensions reduction and deconvolution of differentiating adipocytes gene expression. Gene expression of MDI-induced 3T3-L1 pre-adipocytes (*n* = 98) and human primary adipocytes (HPP) (*n* = 24) was determined by RNA-Seq or microarrays at different times. **(A)** Euclidean distances among samples were calculated from the transformed gene counts. Samples were laid out in two dimensions to approximate the distances and colored by stage (S0, S1, or S2) for non-induced, early or, late differentiation. **(B)** The scatter simplex of the cell mixture is a rotated and compressed version of the scatter simplex of the pure sub-populations. In two dimensions, the sub-populations (P1, P2, and P3) and their markers reside at the extremities. **(C)** Using these markers, the relative fractions of the sub-populations in the mixture of differentiating 3T3-L1 were estimated by standardized averaging. **(D)** The relative fractions of the sub-populations in the mixture of differentiating HPP were estimated by standardized averaging of the same markers.

Averaging the gene expression in a given sample reflects the dominant phenotypes of cells during the course of differentiation. Pre-adipocytes were dissimilar to early and late-differentiated cells. We used as a measure of dissimilarity the distances among the samples within and in-between stage. The distances within the pre-adipocytes samples differed from early (*t* = 24.07; *P* < 0.01) and late-differentiated (*t* = 30.05; *P* < 0.01) samples. Moreover, the stage of differentiation explained about half the variance among the samples (Figure 2A). Un-differentiated and differentiating cells separated across the first two dimensions (*k* = 2; goodness-of-fit > 0.47) of the pairwise distances of the corresponding samples.

The 3T3-L1 cell cultures before and after the induction of differentiation consist of mixtures of cells. Average expression suffices to study the changes in gene expression over time, but the signal of each gene is potentially a composite of its expression in two or more cell types. These may differ in their phenotypes and/or genotypes. At each stage/time point of differentiation, the composition of the culture may differ, and the fractions of the sub-populations may change. Deconvoluting the gene expression of differentiating adipocytes would help address these points.

**Figure 3:**
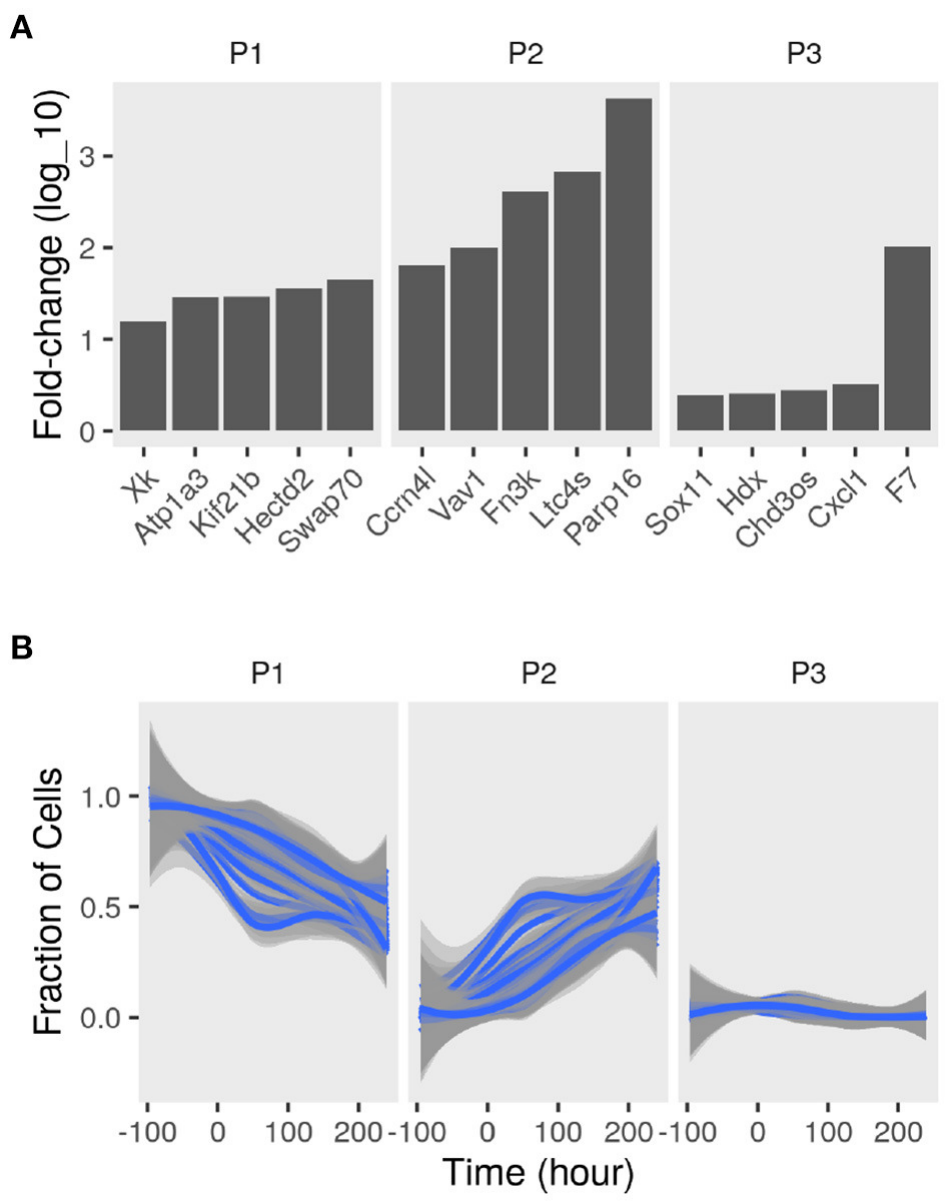
Sub-populations gene markers and fractions in differentiating adipocytes. Gene expression of MDI-induced 3T3-L1 pre-adipocytes (*n* = 98) was determined by RNA-Seq at different time points. Convex analysis of mixture was used to estimate the relative fractions of sub-populations cultures and their specific expression profiles. **(A)** The one-vs-everyone fold-change (log_1_0) was calculated for all genes. The fold-change of the top 5 genes in each sub-populations (P1, P2, and P3) is shown. **(B)** Sets for sub-population markers (set size = 35; iteration number = 100) were re-sampled from the originally identified gene markers. The markers sets were used to re-estimate the relative fractions of the sub-populations (P1, P2, and P3) in the mixture were by standardized averaging. Average fractions are shown as smoothed lines (blue), and the standard errors are shown as ribbons (gray).

To determine the sub-populations in the cultures, we applied the convex analysis of mixtures (CAM) to the mixed gene expression profiles of differentiating adipocytes. The scatter simplex of the expression of the mixture is a transformed version of that of the pure sub-populations. When projected in low-dimensions, we observed three distinct archetypes characteristic of at least three groups of cells (Figure 2B). Gene markers of these sub-populations were identified as genes that are differentially expressed in one sub-populations vs. others (fold-change >1; bootstrapped lower bound confidence interval >0) (Figure 3A and Supplementary File 1). Markers were used to estimate the relative fractions and expression profiles of the subpopulations.

Various sets of sub-population markers gave patterns not very different from the above. We chose random sets of markers of the same size for the three sub-populations (bootstrapping). Lowering the number of markers and randomly re-sampling from the original set gave similar estimates of the fractions of the three populations at most time points (coefficient of variation <10%; bias <0.1) (Figure 3B).

### 3.2. Mature Adipocytes Originate From a Small Fraction of Pre-adipocytes

The starting cell culture is composed of disproportionate fractions of phenotypes that change with time (Figure 2C). One of the three groups (P1) makes up over 95%, and the rest is a mixture of the two other groups. Over the course of differentiation, the fraction of cells of the dominant group decreases to less than 40% of the late samples. In response to the induction stimulus, two factions of cells increase their share of the culture initially. Only one group (P2) continues to increase in size until the end and reaches more than 60% of the cells.

**Figure 4:**
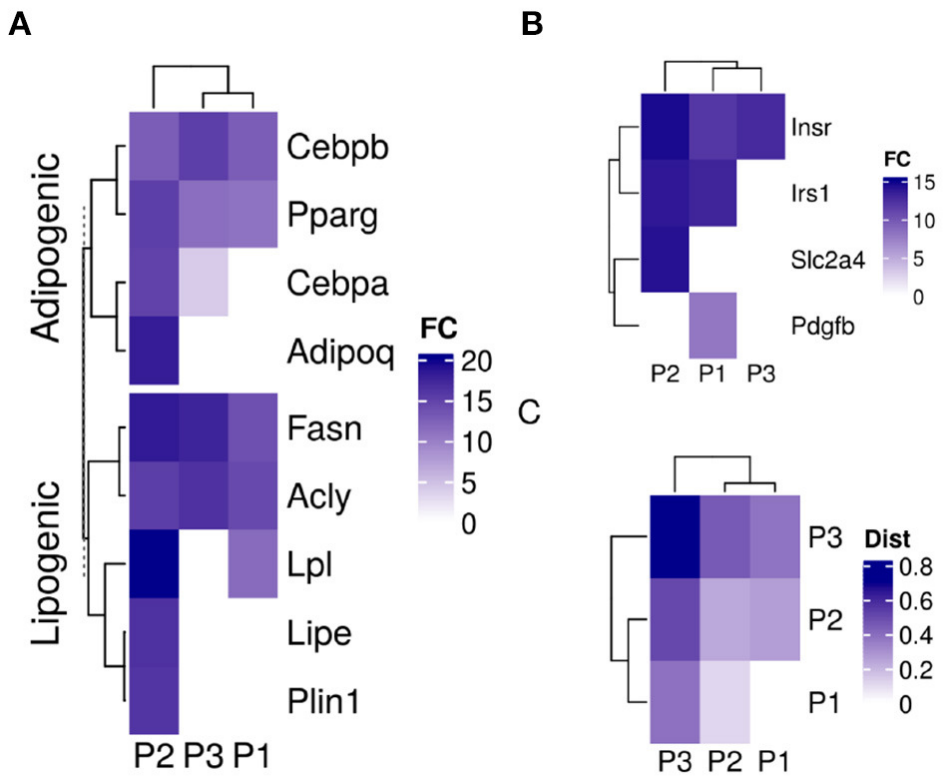
Sub-population specific gene expression in differentiating adipocytes. Gene expression of MDI-induced 3T3-L1 pre-adipocytes (*n* = 98) was determined by RNA-Seq at different time points. Convex analysis of mixture was used to estimate the relative fractions of sub-populations in cultures. The estimated fractions were used to deconvolute the mixed expressions into sub-populations specific profiles by least-square regressions. **(A)** The expression of adipogenic and lipogenic gene markers is shown in three sub-populations. **(B)** The expression of insulin signaling genes is shown in the three sub-populations. **(C)** Spearman's rank correlation coefficient (*r*_*s*_) among the gene expression in the sub-populations of differentiating 3T3-L1 and primary human adipocytes (HPP) were calculated and shown as color values (white, low; blue, high). Sub-populations are indicated as (P1, P2, and P3) and expression as color values (white, low; blue, high).

The three sub-populations were identified based on their specific gene expression profiles. Adipogenic and lipogenic genes marked the second sub-population (P2) as the ones responding to the induction stimulus and forming mature cells (Figure 4A). The first group of genes included the transcription factors *Pparg* and *Cebpb* involved in regulating the differentiation process. Those were highly expressed in P2 compared to the two other sub-populations (fold-change > 7; lower confidence interval > 0). The second group of genes code for key enzymes such as *Fasn* and *Lpl* in the lipid synthesis pathway and are essential for the accumulation of lipid. Those were also expressed at a higher level in P2 (fold-change > 1.5; lower confidence interval > 0).

The differences in gene expression between the two sub-populations extended to more than adipogenic and lipogenic related genes. Insulin metabolism-related genes were different expressed in the differentiating sub-population (P2) compared to the other two (fold-change > 3; lower confidence interval > 1.7) (Figure 4B). Although insulin receptor (*Insr*) and its substrate (*Irs1*) were expressed in the two major sub-populations, the glucose transporter solute carrier family 2 member 4 *(Slc2a4)* was expressed in P1 only. Platelet-derived growth factor subunit B (*Pdgfb*) exclusive expression in P2 indicates non-responsive cells to glucose.

A similar mixture of sub-populations constituteed the cell culture of human primary pre-adipocytes. The fractions of primary cells corresponding to the previously identified markers resembled those estimated from 3T3-L1 cultures (Figure 2D). One sub-populations (P2) grew to dominate the final culture of MDI differentiated cells. The rest (P1 and P3) did not or only briefly responded. Gene expression profiles of the sub-populations in the primary cells were similar to that in the mouse cell model (Figure 4C). Because expression profiles come from two different types of cell lines, the expression of the sub-populations in each were strongly correlated (Spearman's rank correlation coefficient (*r*_*s*_) > 0.6; *P* < 0.001). At the same time, the corresponding sub-populations were strongly correlated between the two cell lines. This was the case for P1 (*r*_*s*_ = 0.47; *P* < 0.001) and P2 (*r*_*s*_ = 0.4; *P* < 0.001). The only exception was the weaker correlations of P3 (*r*_*s*_ = 0.2) which was less than that of the mouse P3 with human P1 (*r*_*s*_ = 0.4; *P* < 0.001).

### 3.3. Sub-populations of Mature Adipocytes Respond Discrepantly to Various Compounds

If, in fact, the mature adipocytes are a composite of different sub-populations, we expect to see a heterogeneous response to the treatment of different compounds. We collected data from MDI-induced adipocytes after day 7 and treated them with various compounds. These compounds included three thiazolidinediones (TZD), four short-chain fatty acids (SFA), and three nonsteroidal anti-inflammatory drugs (NSAID). The response of the cell mixture was evaluated by the regular differential expression, and that of the sub-populations was evaluated by population-specific expression analysis (PSEA). By comparing the response in the adipocyte mixture to that of the individual sub-populations, we could identify the effect of each compound on the mature adipocytes specifically.

**Figure 5:**
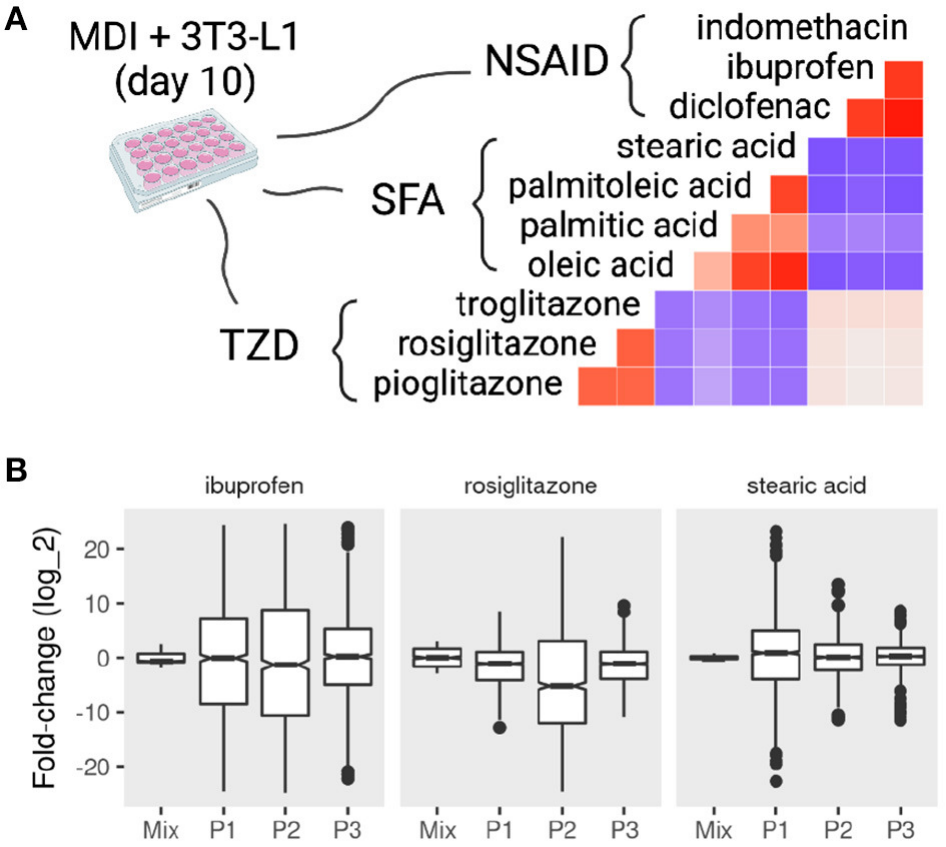
Mixture and sub-population specific response of mature adipocytes to pharmacological perturbations. MDI-induced 3T3-L1 mature adipocytes (> 7 days) were treated with dimethyl sulfoxide (DSMO), nonsteroidal anti-inflammatory drugs (NSAID), short-chain fatty acids (SFA), or thiazolidinediones (TZD). Gene expression of the samples (*n* = 42) was determined by microarrays. Previously identified sub-populations (P1, P2, and P3) specific gene markers in the 3T3-L1 cells were used to estimate the sub-population specific expression profiles. **(A)** The correlations between the fold-change in response to treatments are shown as color values (blue, negative; red, positive). **(B)** The distribution of the fold-change of the top 500 differentially expressed genes in the mixture and sub-populations in response to rosiglitazone, ibuprofen, and stearic acid.

We found that compounds from the same family elicited similar responses as expected (Figure 5A). The response to TZD: rosiglitazone, pioglitazone, troglitazone on the average gene expression was highly correlated (Pearson correlation coefficient (*r*) > 0.5; *P* < 0.001). Similarly, the fold-change in response to SFA and NSAID treatment was correlated (*r* > 0.4 and *r* > 0.6; *P* < 0.001). Only small correlations were found between the fold-change response between groups of compounds. TZD treatments were positively correlated with the NSAID and negatively correlated with that of SFA. NSAID and SFA were negatively correlated.

The response on the sub-population level to the treatment of different compounds often varied from the mixture and among each other (Figure 5B and Supplementary File 2). Treatment with rosiglitazone produced more pronounced differential expression in P2 compared to the other populations (KS test *D*^−^ < 0.15; *P* < 0.001) as well as the mixture (*D*^−^ < 0.24; *P* < 0.001). A similar pattern was observed with the treatment of ibuprofen (*D*^−^ < 0.13; *P* < 0.001). By contrast, P1 was more different when adipocytes were treated with stearic acid (*D*^−^ = 0.43; *P* < 0.001).

### 3.4. A Dynamic Model of Two Sub-populations in a Fully Confluent Cell Culture

**Figure 6:**
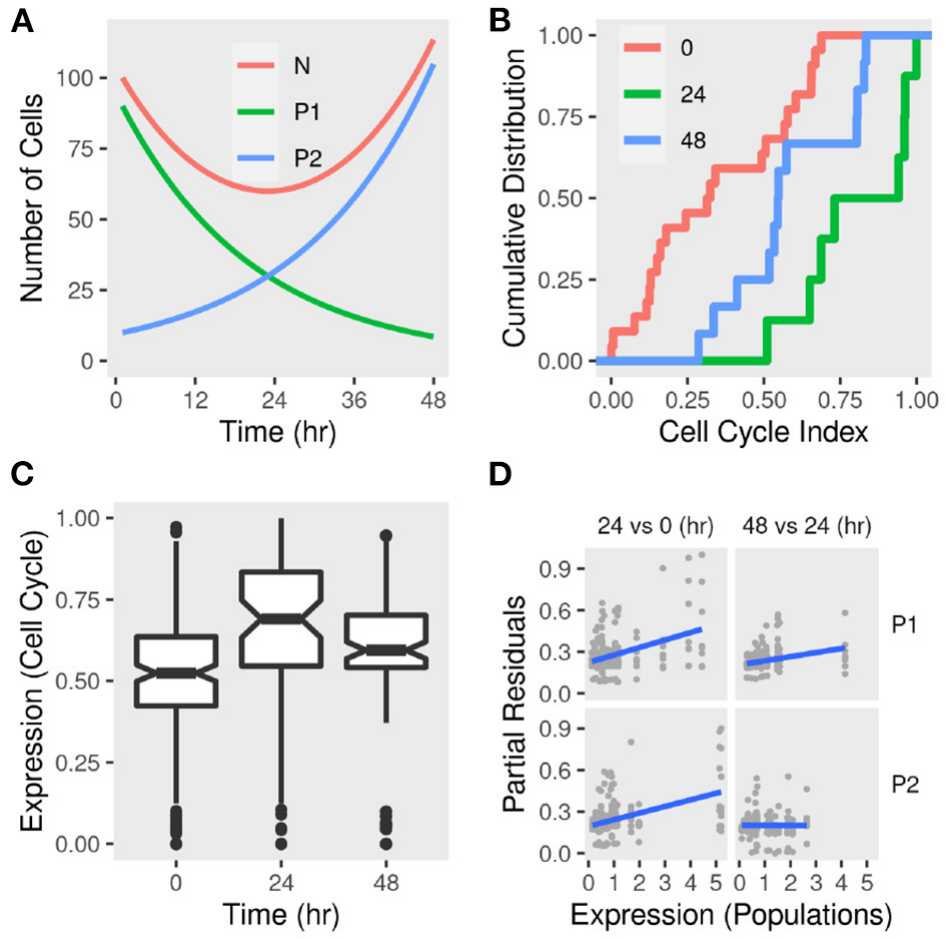
Sub-population dynamics and cell cycle gene expression signature in differentiating adipocytes. **(A)** A toy model of two sub-populations *P*1 and *P*2 growing at rates −*r* and *r*, respectively and the total population *N*. The change in the total number of cells and each sub-population was calculated over time given reasonable initial states and parameter values. **(B)** Gene expression of MDI-induced 3T3-L1 pre-adipocytes (*n* = 42) was determined by RNA-Seq at three time points. The cumulative distribution function of eight-cell cycle genes in differentiating adipocytes at 0, 24, and 48 h. **(C)** The distribution of the expression of eight-cell cycle genes in differentiating adipocytes at the same time points. **(D)** Previously identified sub-populations (P1 and P2) specific gene markers were used to estimate the sub-population specific expression profiles. Regression models were used to estimate the differential expression between 24 and 0 h and between 48 and 24 h. Partial residuals of each comparison and population-specific expression are shown.

To show the validity of these observations, we constructed a model of sub-populations dynamics and compared it with further observations from the data. In this model, two sub-populations of different sizes grow at different rates in response to the environment in a fully confluent cell culture (Figure 6A). *P*2 is a small sub-population that grows at a rate *r*, *rP*1 while *P*1 is a large sub-population that decreases in size at a smaller or the same rate −*r*, −*rP*1. In this case, the total population size (*N*) can be estimated as (*P*2 − *P*1)*r*. This predicts that the total population (*N*) should decrease initially and bounces to the same size or higher after 2 days, given a small *r*. This system can be summarized as follows:

(1) $$\begin{array}{r} \frac{dP1}{dt}=-rP1 \end{array}$$

(2) $$\begin{array}{r} \frac{dP2}{dt}=rP2 \end{array}$$

(3) $$\begin{array}{r} \frac{dN}{dt}=\left(P2-P1\right)r \end{array}$$

A fully confluent cell culture imposes a constraint on the rate at which the sub-populations can grow or die (*r*). We chose a value that re-establishes the population size within a reasonable period (2 days). The rate at which one population can grow is bound by the rate at which the size of the other population shrinks. The values of the initial states (*P*1, *P*2, and *N*) were suggested by the previous analysis where the growing sub-population represents a small fraction of the total at the start of the cell culture.

### 3.5. Cell Cycle Index Fits the Dynamics of Two Sub-populations

We used a gene expression signature (*Mki67, Rb1, Hist1h2ae, Ccnb1, Cbx3, Gapdh, Ccnb2, and E2f1)* to estimate the cell cycle phase at population and sub-population levels (Dolatabadi et al., 2017). We found that, adipocytes at day 1 had higher cell cycle indecies than pre-adipocytes (KS test *D*^−^ = 0.7; *P* < 0.01) and adipocytes at day 2 (*D*^−^ = 0.5; *P* > 0.05) (Figure 6B). This pattern reflects the higher expression of cell cycle genes at day 1 compared to pre-adipocytes (enrichment score = 0.7; *P* < 0.05) and adipocytes at day 2 (enrichment score = 0.6; *P* > 0.05) which indicates a larger proportion of the cells in G0/S phase and, therefore, less active divisions (Figure 6C). This was consistent with the predicted population size of the toy model.

The sub-population specific differential expression confirmed that the two populations contribute differently to the expression of cell cycle genes and therefore to the active cell division (Figure 6D). This is clear from the correlation between the expression of the cell cycle genes and the reference (population signals). It is not clear from this analysis how to assign specific values to each sub-population at each of the two comparisons. This is because the expression is calculated for each of the eight genes individually. Moreover, the index reflects a continuous transition from G1 to S phase of the cell cycle rather than discrete categories. This would be affected by the size of the population at different time points and, therefore, their contribution to the overall expression of the genes constituting the index.

### 3.6. Resistance to Differentiation May Be Due to Structural Restrictions on the Cell and/or Commitment to Other Lineages

Several functional sets of genes were over-represented (count ≥ 3; false-discovery rate < 0.2) in the group of markers of the growing sub-population (P2). These were related to fat differentiation, nuclear receptors and, transcriptional activities (Table 3). More specifically, many of the characteristic markers were part of the process of *brown fat cell differentiation*. Molecular functions such as *nuclear receptor* and *steroid hormone activities* were over-represented in the same group. Several of these markers localized to cellular components such as *transcription factor complex*. Alternatively, the phenotype that did not show these characteristics could be thought of as resisting the induction stimulus. Indeed, completely different processes and functions were over-represented by the markers of the other two populations.

**Table 3:** Gene set over-representations in the sub-populations specific gene markers.

**Pop**.	**Onto**.	**Term**
P1	BP	establishment of mitotic spindle orientation
		actin filament bundle assembly
		actin cytoskeleton organization
		regulation of cell shape
		regulation of cell adhesion
	CC	spindle pole
		microtubule organizing center
		actin filament
		cell-cell junction
		bicellular tight junction
		actin cytoskeleton
	MF	actin binding
		actin filament binding
		transcription regulatory region DNA binding
		muscle alpha-actinin binding
P2	BP	actin cytoskeleton organization
		negative regulation of Rho protein signal transduction
		brown fat cell differentiation
	CC	stress fiber
		RNA polymerase II transcription factor complex
	MF	nuclear receptor activity
		steroid hormone receptor activity
		nuclear receptor transcription coactivator activity
P3	BP	positive regulation of gene expression
		neuron differentiation
		keratinocyte differentiation
		keratinization
	CC	cornified envelope
		extracellular matrix
	MF	signaling receptor binding
		structural molecule activity
		structural constituent of cytoskeleton

The markers of the other sub-populations were enriched in terms related to cellular structural processes and components (Table 3). The first group (P1) had multiple markers related to *mitotic spindles, microtubules, actin* and *cytoskeleton*. All are essential components of the cell structure and necessary for allowing morphological changes. In addition to these terms, several processes related to the assembly and organization of these components were enriched by P1 markers.

The third group (P3) had terms related to structural components, namely the *extracellular matrix* and molecular functions *structural constituent of cytoskeleton* enriched by its markers. But also biological processes terms related to the differentiation of cell types other than adipocytes, *neurons* and *keratinocytes differentiation* were over-represented in the P3 set of markers.

## 4. Discussion

**Figure 7:**
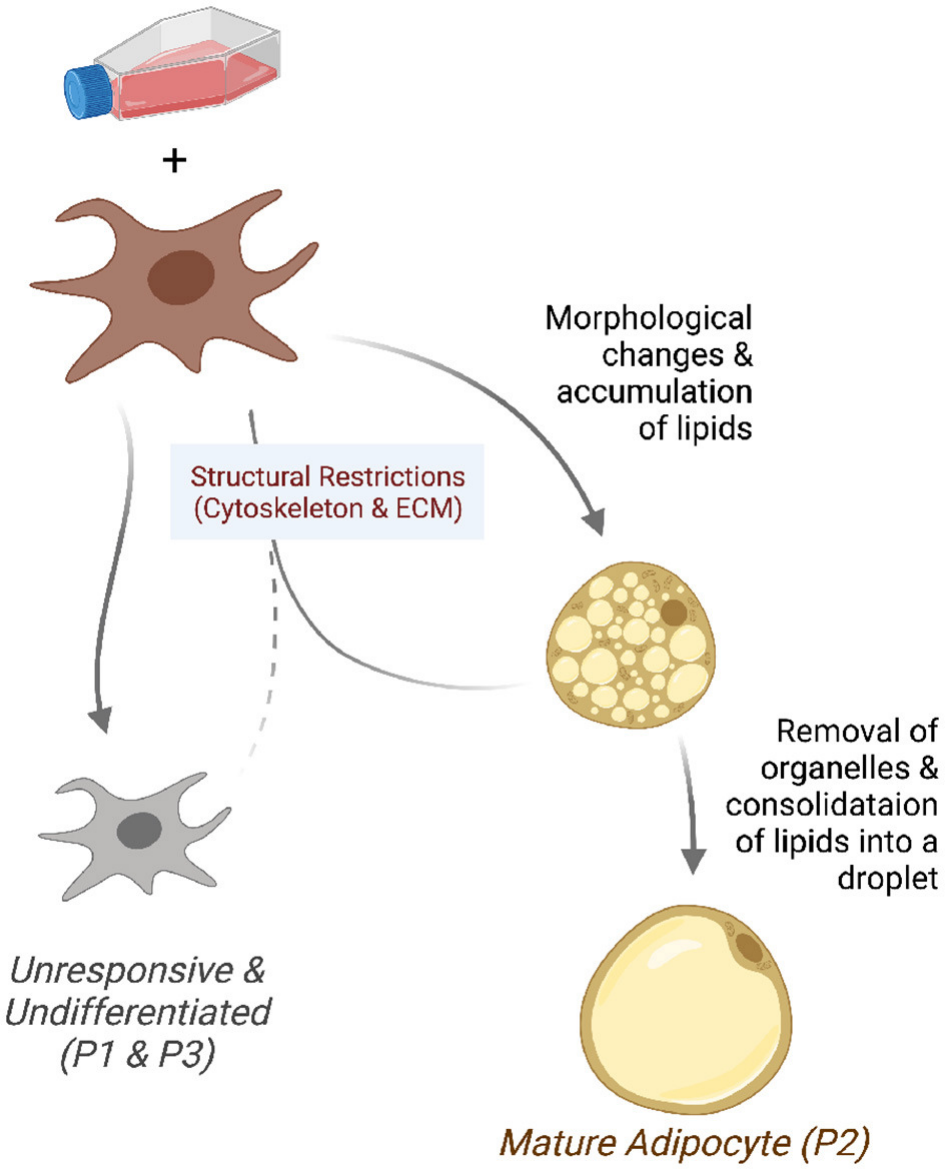
Model of the differentiating adipocyte sub-populations. A fraction of pre-adipocytes growing in differentiation media responds to adipogenic stimuli. Responsive cells (P2) undergo morphological changes and accumulate lipids into small droplets. Adipocytes reach maturity after intracellular organelles are removed and fat consolidated into bigger droplets. Cells that fail to overcome structural restrictions (P1 and P3) such as those in the cytoskeleton and extracellular matrix do not undergo morphological changes and remain undifferentiated.

We found that the pre-adipocyte cell culture is composed of three distinct sub-populations. After the differentiation induction, a minority of cells grows and transforms into mature adipocytes. This fraction of cells have a gene expression profile conducive to activities related to the nuclear receptors, fat differentiation, and gene transcription. The sub-populations of cells that do not respond to induction are under structural constraints or are committed to differentiate into other cell types. Figure 7 summarizes these observations in a graphical representation, which we further discuss below.

Identifying the factors that render fibroblasts responsive or resistant to the induction medium would help achieve better differentiation efficiency. More importantly, it would improve our understanding of adipose tissue composition and how to encourage or discourage the terminal maturation of the adipocytes.

In the context of CAM, exclusively enriched genes in a subset of cells are the markers for the set (Wang et al., 2016). The expression of other genes (co-expressed) is the combination of their expression in the sub-populations. The expression of all genes in the mixture forms a k-dimensional simplex with a telling geometry. The simplex shape encloses the data, and the sub-populations with their markers are at the extremes.

To demonstrate the validity of these findings, one has to show that (1) pre-adipocyte cell cultures consist of distinct populations and that (2) some cells but not others can increase their population size. Differences intrinsic to the cells, in the ability to respond to MDI, in the kind of cell they become, or a combination of the above could give rise to the observed pattern. Although the size of the sub-populations is dissimilar to start with, it only changes after the differentiation induction. Because only the fractions of each sub-population rather than their absolute size were estimated, the inflection is possible to achieve with either the duplication of the responsive cells or the shrinkage of the others. Indeed, pre-adipocytes can replicate after the induction (Bernlohr et al., 1985). Mature adipocytes can divide and redistribute the lipid droplets between the daughter cells (Nagayama et al., 2007).

Previous studies pointed out the heterogeneous nature of adipocyte cell cultures and adipose tissue progenitors. Often these studies focused on the phenotypic and molecular differences among the identified populations. Most attribute these differences to causes in two categories: asynchronous differentiation and lineage commitment. The first refers to cells that are essentially similar but only take longer to differentiate (Loo et al., 2009). Therefore, at any specific point, some cells would lack the characteristics of mature adipocytes. For example, poor-lipid accumulating cells would form larger droplets of lipid by merely prolonged simulation (Le and Cheng, 2009). While our study does not rule out this possibility, it makes counter observations. First, the differences between the sub-populations are stark from the very beginning (pre-adipocytes). Second, the lagging sub-populations do not show any sign of differentiation either in adipogenic regulation or lipid synthesis. Finally, the dataset extends up to 2 weeks, twice the period of typical maturation.

Studies based on the adipose tissue progenitor cells suggest that some cells are either incapable of differentiating into mature adipocytes, originate in certain depots, or can only differentiate into other cell types (brown/beige) adipocytes. One study suggested that mouse visceral, but not subcutaneous, white adipose tissue arises from cells expressing Wilms tumor (*Wt1*) gene (Chau and Hastie, 2014). Another found that the adipocyte's ability to accumulate fat is heritable (Katz et al., 2014). Histone modification of PPARG promoter marked cells which can de-differentiate and re-differentiate. It's even been shown that a minority sub-population lacking the typical adipocyte features might take on a regulatory role to influence its surroundings in a paracrine manner (Schwalie et al., 2018).

Here, we suggest that the mature adipocytes arise from a minority of the pre-adipocytes. Most pre-adipocytes cannot differentiate properly or accumulate lipids. This lack of responsiveness to the differentiation medium is either due to or associated with structural restraints. The major factors that differentiate the two types of cells are genes involved in the cellular cytoskeleton and actin filaments. Molecular differences also exist, most importantly in regulating adipogenesis, lipid synthesis, and insulin signaling.

This study was limited to publicly available gene expression data of the 3T3-L1 cell model and primary adipocytes. Experimental validation is required to identify the role of individual protein markers, their distributions in the sub-populations, and the cellular processes they are involved in. For example, would manipulating the proteins involved in organizing the cell structure or extracellular matrix affect the cell's ability to differentiate? These experiments should be done on single cells or separate the populations based on their potential to differentiate, which we plan to do in future investigations.

## Data Availability Statement

The data analyzed in the study are deposited in Figshare, https://doi.org/10.6084/m9.figshare.9906182 and https://doi.org/10.6084/m9.figshare.13088624.

## Author Contributions

MA conceived, performed, and reported the analysis. TL helped with the conceptualization and the writing of the manuscript. DK acquired the funding, supervised, and contributed to the concepts and the writing of the manuscript. All authors contributed to the article and approved the submitted version.

## Funding

This study was supported by the National Research Foundation of Korea (NRF) grant funded by the Ministry of Science and ICT (MSIT) of the Korea government (2015R1A5A2008833 and 2020R1A2C2011416).

## Conflict of Interest

The authors declare that the research was conducted in the absence of any commercial or financial relationships that could be construed as a potential conflict of interest.

## Publisher's Note

All claims expressed in this article are solely those of the authors and do not necessarily represent those of their affiliated organizations, or those of the publisher, the editors and the reviewers. Any product that may be evaluated in this article, or claim that may be made by its manufacturer, is not guaranteed or endorsed by the publisher.
